# Steatotic liver-derived extracellular vesicles deliver ACSL4 to potentiate hepatic ischemia–reperfusion injury by orchestrating a ferroptosis-mitochondrial apoptosis cascade

**DOI:** 10.1016/j.gendis.2025.102005

**Published:** 2025-12-24

**Authors:** Hangcheng Zhao, Jialing Zhao, Danfeng Zhou, Yunguo Lei, Zhikun Liu, Jun Chen, Peiyang Hu, Xiao Xu, Haiyang Xie, Qiang Wei

**Affiliations:** a Institute of Translational Medicine, Zhejiang University School of Medicine, Hangzhou, Zhejiang 310029, China; b NHC Key Laboratory of Combined Multi-organ Transplantation, Institute of Organ Transplantation, Zhejiang University, Hangzhou, Zhejiang 310003, China; c Zhejiang Chinese Medical University, Hangzhou, Zhejiang 310053, China; d School of Clinical Medicine, Hangzhou Medical College, Hangzhou, Zhejiang 310053, China; e Department of Hepatobiliary & Pancreatic Surgery and Minimally Invasive Surgery, Zhejiang Provincial People's Hospital, Affiliated People's Hospital, Hangzhou Medical College, Hangzhou, Zhejiang 310014, China; f Department of Traumatology, Tiantai People's Hospital of Zhejiang Province (Tiantai Branch of Zhejiang People's Hospital), Hangzhou Medical College, Taizhou, Zhejiang 317200, China

**Keywords:** ACSL4, Extracellular vesicles, Ferroptosis, Hepatic ischemia–reperfusion injury, Mitochondrialapoptosis, Steatotic liver graft

## Abstract

Steatotic liver grafts exhibit heightened susceptibility to hepatic ischemia–reperfusion injury (HIRI), contributing to poor outcomes in liver transplantation; however, the underling mechanisms remain incompletely defined. Here, we demonstrate that extracellular vesicles derived from steatotic livers (HFD-EVs) exacerbate HIRI by delivering acyl-CoA synthetase long-chain family member 4 (ACSL4) to hepatocytes, thereby triggering a ferroptosis-mitochondrial apoptosis cascade. Proteomic profiling revealed that HFD-EVs are enriched with ACSL4 and other pro-ferroptotic mediators, but deficient in mitochondrial oxidative phosphorylation components. In a murine model, administration of HFD-EVs to lean mice prior to ischemia–reperfusion significantly aggravated liver injury, apoptosis, and inflammatory responses compared to EVs from healthy livers. *In vitro*, HFD-EVs were internalized by hepatocytes, leading to ACSL4 up-regulation, GPX4 suppression, and subsequent iron-dependent lipid peroxidation. This ferroptotic stress induced mitochondrial calcium overload, permeability transition pore opening, membrane depolarization, and ultrastructural damage, culminating in cytochrome c release and apoptotic cell death. Critically, inhibition of ferroptosis with Ferrostatin-1 abolished ACSL4 induction, restored mitochondrial integrity, and rescued cell viability, confirming the central role of ferroptosis in HFD-EVs-induced injury. Our study unveils a previously unrecognized mechanism by which HFD-EVs transmit ACSL4 to initiate ferroptosis and mitochondrial dysfunction in recipient hepatocytes, providing a mechanistic basis for the vulnerability of steatotic grafts to HIRI. These findings highlight HFD-EVs and the ACSL4-ferroptosis axis as promising therapeutic targets to improve the quality of fatty liver grafts in transplantation.

## Introduction

Hepatic ischemia–reperfusion injury (HIRI) is a major complication of liver transplantation and resection, significantly contributing to post-operative graft dysfunction and failure.^1^^,^^2^ The pathological cascade of HIRI involves a complex interplay of metabolic stress, inflammatory responses, and various modes of hepatocellular death.^3^ The severity of HIRI is critically influenced by the underlying condition of the liver. With the worldwide surge in metabolic dysfunction-associated steatotic liver disease (MASLD), the use of steatotic donor livers has become increasingly common.^4^^,^^5^ However, these grafts are markedly more susceptible to ischemia–reperfusion injury (IRI), leading to higher rates of primary non-function and poor clinical outcomes.^6^ Understanding the mechanisms behind this heightened vulnerability is therefore of paramount clinical importance.

Accumulating evidence underscores the role of extracellular vesicles (EVs) as key mediators of intercellular communication in liver pathophysiology.^7^, ^8^, ^9^ Released by virtually all cell types, EVs transport bioactive molecules, including proteins, lipids, and RNAs, to recipient cells, thereby modulating tissue homeostasis and disease progression. In metabolic liver diseases, the altered cellular microenvironment is likely reflected in the composition and function of secreted EVs.^10^ It is plausible that steatotic hepatocytes release EVs with a distinct cargo that can prime the liver for exacerbated injury upon ischemic insult.^11^ Nevertheless, the specific contribution of steatotic liver-derived EVs (HFD-EVs) to HIRI, and the molecular mechanisms involved, remain poorly defined.

Among the mechanisms of liver injury, regulated cell death pathways are critically implicated in HIRI. Beyond classical apoptosis, recent attention has focused on ferroptosis, an iron-dependent form of cell death characterized by uncontrolled lipid peroxidation.^12^ The steatotic liver, with its abundance of peroxidation-susceptible polyunsaturated fatty acids and altered redox balance, may provide a fertile ground for ferroptosis activation upon ischemia–reperfusion stress. Furthermore, mitochondrial dysfunction is a hallmark of both HIRI and steatotic liver disease, often culminating in the permeabilization of the outer mitochondrial membrane and the activation of the intrinsic apoptosis pathway.^13^ While these injury modalities—ferroptosis and mitochondrial apoptosis—have been studied individually, their potential interplay in the context of steatotic liver transplantation, and whether EVs serve as a connecting link, are intriguing but unanswered questions.

Based on these considerations, we hypothesized that HFD-EVs may carry a pathogenic cargo that sensitizes hepatocytes to HIRI by orchestrating specific cell death pathways. This study aimed to systematically investigate the impact of HFD-EVs on HIRI severity and to delineate the underlying mechanisms, with a particular focus on regulated cell death processes and mitochondrial integrity.

## Methods and materials

### Animal model and experimental procedures

C57BL/6 male mice (8 weeks old, weighing 20 ± 2 g) were obtained from the Experimental Animal Center of Zhejiang Academy of Medical Sciences (Hangzhou, China). All experimental protocols were conducted in accordance with the institutional ethical guidelines and approved by Institutional Animal Care and Use Committee of Zhejiang Center of Laboratory Animals (ZJCLA-IACUC-200011061). Animals were maintained under specific-pathogen-free (SPF) conditions at a controlled temperature of 22 ± 1 °C, relative humidity of 50%, and a 12-h light/dark cycle. Mice were randomly allocated to receive either standard laboratory chow or high-fat diet (HFD; 60% kcal from fat, D12492, Changzhou Mouse Biotech Co., Ltd., China) for 12 weeks to induce hepatic steatosis. Water and food were provided ad libitum throughout the experimental period.

The HIRI model was established through total hepatic ischemia. Briefly, mice were anesthetized with 2% isoflurane (RWD Life Science, China) in oxygen. After midline laparotomy, the hepatic portal triad (portal vein and hepatic artery) was exposed. Total hepatic ischemia was induced by clamping the portal vein and hepatic artery at the hepatic hilum using a non-traumatic microvascular clamp (RWD Life Science, China) for 60 min. Reperfusion was initiated by clamp removal, confirmed by visible restoration of hepatic blood flow. Sham-operated controls underwent identical procedures without vascular occlusion.

For experimental interventions, mice were randomly allocated to four groups: sham-operated controls undergoing vascular exposure without clamping (sham group), IRI models receiving phosphate buffer saline (PBS) injection (NC IRI group), IRI models administered EVs from normal chow-fed mice (NC-EVs + IRI group), and IRI models treated with EVs from high-fat diet-fed mice (HFD-EVs + IRI group). All EVs were isolated from liver tissues via differential ultracentrifugation and standardized to 20 mg/kg in PBS. EV suspensions or PBS vehicle were administered through tail vein injection 24 h prior to HIRI induction. At 6 h post-reperfusion, the animals were euthanized by cervical dislocation under deep anesthesia. Blood samples were collected via cardiac puncture for serum biochemical profiling. Liver tissues were systematically processed with the left lateral lobe fixed in 4% paraformaldehyde for histological evaluation, while the median lobe was immediately snap-frozen in liquid nitrogen for subsequent protein analyses.

### Cell culture, hepatocyte isolation, and experimental treatments

AML12 murine hepatocytes (Procell, China) and primary hepatocytes isolated from C57BL/6 mice were utilized for *in vitro* experiments. Cells were maintained in complete growth medium consisting of DMEM/F12 (Gibco, USA) supplemented with 1% ITS (Insulin-Transferrin-Selenium, Gibco, USA, #41400045), 40 μg/mL dexamethasone (Sigma–Aldrich, USA, #50-02-2), and 10% fetal bovine serum (Zhejiang Baidi Biotechnology, China, #F814-500). Cultures were incubated at 37 °C in a humidified atmosphere containing 5% CO_2_, with medium replacement every 48 h.

Primary hepatocytes were isolated using a two-step collagenase perfusion technique. Briefly, mice were anesthetized by intraperitoneal injection of sodium pentobarbital (50 mg/kg), followed by portal vein cannulation and perfusion with calcium-free Hank's Balanced Salt Solution (HBSS) at 37 °C. Subsequently, the liver was digested with 0.05% collagenase type IV (Sigma–Aldrich, USA, #C5138) in HBSS containing 5 mM CaCl_2_. The resulting cell suspension was filtered through a 70-μm cell strainer and purified by Percoll (Sigma–Aldrich, USA, #P4937) density gradient centrifugation (50% v/v, 800 × *g*, 15 min). Viable hepatocytes were seeded onto collagen-coated culture dishes and allowed to attach for 4 h before medium replacement.

To establish the *in vitro* ischemia–reperfusion injury model, cells were pretreated with NC-EVs or HFD-EVs (100 μg/mL), HFD-EVs (100 μg/mL) + Ferrostatin-1 (Fer-1, 2 μM, MedChemExpress, USA, #HY-100579), or PBS vehicle control for 24 h. Hypoxic conditions were induced using a tri-gas incubator (Thermo Fisher Scientific) maintained at 1% O_2_, 5% CO_2_, and 94% N_2_ for 12 h, followed by reoxygenation under normoxic conditions (21% O_2_) for an additional 12 h.

### Biochemical analysis of hepatic injury markers

Following blood collection via cardiac puncture, serum samples were obtained by centrifugation at 3000 × *g* for 15 min at 4 °C. Hepatic injury was quantified by measuring serum levels of alanine aminotransferase (ALT), aspartate aminotransferase (AST), and lactate dehydrogenase (LDH) using an automated biochemical analyzer (Mindray BS-800M, China) according to the manufacturer's protocols. All measurements were performed in triplicate, and the results were normalized to sham-operated controls.

### RNA extraction, reverse transcription, and quantitative PCR (qPCR)

Total RNA was extracted from tissues or cells using the Animal Total RNA Isolation Kit (ABclonal, China, #RK30120) according to the manufacturer's instructions. The concentration and purity of RNA were determined using a NanoDrop spectrophotometer. cDNA was synthesized from 1 μg of total RNA using the ABScript II cDNA First-Strand Synthesis Kit (ABclonal, China, #RK20400). Quantitative PCR (qPCR) was performed using 2 × Universal SYBR Green Fast qPCR Mix (Abclonal, China, #RK21203) on a QuantStudio 6 Pro system (Thermo Fisher, USA). Relative gene expression was calculated using the 2 ^^^–ΔΔCt method with ACTIN as an internal control. All primers used are listed in Supplementary Table S1.

### Protein quantification by immunoblot analysis

Proteins were extracted using RIPA lysis buffer with protease/phosphatase inhibitors, separated via SDS-PAGE, and transferred to PVDF membranes (Millipore, Germany). Membranes were blocked with QuickBlock buffer (Beyotime, China), incubated with primary/secondary antibodies, and washed with TBST. Signals were detected using a chemiluminescence system (ProteinSimple, USA). Antibody details are shown in Supplementary Table S2.

### Cell viability assessment

Cell viability was determined using the Cell Counting Kit-8 (MedChemExpress, USA) according to the manufacturer's protocol. Briefly, AML12 cells and primary hepatocytes were seeded in 96-well plates at a density of 1 × 10^4^ cells/well and allowed to adhere for 24 h. Cells were then treated with NC-EVs or HFD-EVs (100 μg/mL) for 24 h, followed by hypoxia-reoxygenation (H/R) injury induction.

After treatment, 10 μL of CCK-8 reagent was added to each well, and plates were incubated at 37 °C for 2 h. Absorbance was measured at 450 nm using a microplate reader (Synergy H1, BioTek, USA). Cell viability was calculated as the percentage of absorbance relative to untreated controls. All experiments were performed in triplicate and repeated three times independently.

## Oxidative stress and apoptosis analysis

### Reactive oxygen species (ROS) detection

Intracellular ROS levels were measured using the DCFH-DA fluorescent probe (Beyotime, China, #S0033S) coupled with fluorescence microscopy and flow cytometry. Following H/R injury induction, cells were incubated with 10 μM DCFH-DA in serum-free medium at 37 °C for 30 min. After washing with PBS, the cells were processed for parallel analyses. For qualitative assessment, fluorescence images were captured using an Olympus IX73 fluorescence microscope (Olympus, Japan) equipped with FITC filters (Ex/Em = 488/525 nm). For quantitative analysis, cells were trypsinized, resuspended in PBS, and immediately analyzed using a flow cytometer (BD FACSCelesta, BD Biosciences, USA). A minimum of 10,000 events per sample were acquired, and the mean fluorescence intensity (MFI) was quantified using FlowJo software.

### Terminal deoxynucleotidyl transferase dUTP nick-end labeling (TUNEL) assay

Apoptotic cells were detected using the One Step TUNEL Apoptosis Assay Kit (Beyotime, China, #C1089) according to the manufacturer's instructions. Briefly, cells were fixed with 4% paraformaldehyde for 30 min at room temperature, permeabilized with 0.1% Triton X-100 for 10 min, and incubated with TUNEL reaction mixture for 1 h at 37 °C. Nuclei were counterstained with DAPI (1 μg/mL) for 5 min. Fluorescent images were acquired using an Olympus IX73 inverted fluorescence microscope (Olympus, Japan) equipped with a DP80 dual CCD camera system. Apoptotic cells were quantified by analyzing fluorescence intensity with ImageJ software using standardized thresholding protocols.

### Proteomic profiling of EVs

Proteomic analysis of isolated EVs was conducted by Lc-Bio Technologies (Hangzhou, China) using liquid chromatography-tandem mass spectrometry (LC-MS/MS) following established protocols. Briefly, EV protein samples were digested with trypsin (Promega, USA) and analyzed using a Q Exactive HF-X mass spectrometer (Thermo Fisher Scientific, USA) coupled with an EASY-nLC 1200 system (Thermo Fisher Scientific). Raw data were processed using MaxQuant software (version 1.6.17.0) against the UniProt Mus musculus database. Differentially expressed proteins between NC-EVs and HFD-EVs were identified using a fold change threshold of > 2.0 and a false discovery rate (FDR) of < 1%.

Subsequently, functional annotation and pathway enrichment analysis of the differentially expressed proteins were performed using the DAVID bioinformatics platform (version 6.8). To ensure an unbiased and biologically relevant interpretation of the proteomic data, we adopted rigorous criteria for the presentation of Kyoto Encyclopedia of Genes and Genomes (KEGG) pathways. Specifically, from all pathways significantly enriched, we systematically selected those with high relevance to the core biological themes of this study, namely ferroptosis, mitochondrial function (including oxidative phosphorylation), and regulated cell death (*e.g.*, apoptosis).

### Isolation and purification of liver-derived EVs

Liver tissue-derived EVs were isolated from C57BL/6 mice using a modified differential centrifugation protocol. Following anesthesia with sodium pentobarbital (50 mg/kg, i.p.), livers were perfused through the portal vein with 0.05% collagenase type IV in HBSS at 37 °C. The digested tissue was mechanically homogenized using a Dounce homogenizer (Wheaton, USA) and filtered through a 70-μm cell strainer (Corning, USA). The homogenate was subjected to sequential centrifugation steps: 300 × *g* for 10 min to remove intact cells, 2000 × *g* for 10 min to eliminate cell debris, and 10,000 × *g* for 30 min to pellet large vesicles and organelles. The resulting supernatant was ultracentrifuged at 100,000 × *g* for 90 min at 4 °C using an Optima XE-90 ultracentrifuge (Beckman Coulter, USA) with a Type 70 Ti rotor. The EV pellet was washed twice in phosphate-buffered saline (PBS) through additional ultracentrifugation cycles (100,000 × *g*, 90 min) to ensure purity. The final EV preparation was resuspended in sterile PBS, quantified using a bicinchoninic acid (BCA) protein assay (Pierce, USA), and stored at −80 °C until further analysis. The EV size distribution was analyzed using a Zetasizer Nano ZS90 particle size analyzer (Malvern Panalytical, UK) based on dynamic light scattering (DLS) technology.

## *In vivo* and *in vitro* exosome tracking

### *In vivo* EV biodistribution analysis

For *in vivo* tracking, EVs were labeled with the near-infrared fluorescent dye DIR (1,1′-dioctadecyl-3,3,3′,3′-tetramethylindotricarbocyanine iodide; Thermo Fisher Scientific, USA, #D12731) at a 1:1000 dilution in PBS. Following a 10-min incubation at 37 °C, unbound dye was removed by ultracentrifugation (100,000 × *g*, 90 min, 4 °C). DIR-labeled exosomes (100 μg in 200 μL PBS) were administered via tail vein injection into C57BL/6 mice. Whole-body fluorescence imaging was performed at 24 h post-injection using an IVIS Spectrum *in vivo* imaging system (PerkinElmer, USA) under isoflurane anesthesia. Following imaging, mice were euthanized, and major organs (liver, spleen, kidneys, lungs, and heart) were excised for *ex vivo* fluorescence quantification. The fluorescence intensity was analyzed using Living Image software (version 4.7.3, PerkinElmer), with the results normalized to the background signal.

### *In vitro* EV uptake assay

For cellular tracking, EVs were labeled with the lipophilic dye PKH67 (Solarbio, Beijing, China, #IKC10664) at a 1:200 dilution in Diluent C for 5 min at room temperature. The labeling reaction was terminated by adding an equal volume of 1% bovine serum albumin (BSA) in PBS, followed by ultracentrifugation (100,000 × *g*, 90 min, 4 °C) to remove unbound dye. Labeled EVs (100 μg/mL) were incubated with AML12 cells or primary hepatocytes for 24 h at 37 °C. The cells were subsequently fixed with 4% paraformaldehyde and counterstained with 4′,6-diamidino-2-phenylindole (DAPI; Solarbio, #28718-90-3) for nuclear visualization. EV internalization was assessed using a confocal laser scanning microscope (FV3000, Olympus, Japan) with excitation/emission wavelengths of 490/502 nm for PKH67 and 358/461 nm for DAPI.

### Assessment of mitochondrial membrane potential

Mitochondrial membrane potential (ΔΨm) was evaluated using the JC-1 fluorescent probe (MedChemExpress, USA, #HY-15534). AML12 cells were seeded at a density of 1 × 10^5^ cells per well in glass-bottom dishes (NEST Biotechnology, China) and allowed to adhere overnight. Cells were pretreated with NC-EVs or HFD-EVs (100 μg/mL) for 24 h, followed by hypoxia-reoxygenation (H/R) treatment. After treatment, the cells were incubated with 5 μM JC-1 in serum-free medium for 20 min at 37 °C in the dark. Unbound dye was removed by two washes with PBS.

JC-1 exhibits potential-dependent accumulation in mitochondria, forming green fluorescent aggregates (excitation/emission: 585/590 nm) in polarized mitochondria, while remaining as red fluorescent monomers (excitation/emission: 514/529 nm) in depolarized mitochondria. Fluorescence images were acquired using a confocal microscope (FV3000, Olympus) with appropriate filter sets. The ratio of red and green fluorescence intensity was quantified using ImageJ software (NIH, USA) to assess mitochondrial depolarization.

### Mitochondrial morphological analysis using MitoTracker™ staining

Mitochondrial morphology was assessed in AML12 cells and primary hepatocytes using MitoTracker™ Red CMXRos (Beyotime Biotechnology, Shanghai, China, #C1032). Cells were seeded at a density of 1 × 10^5^ cells per well in glass-bottom culture dishes (NEST Biotechnology, China) and allowed to adhere overnight. Following pretreatment with NC-EVs or HFD-EVs (100 μg/mL) for 24 h, cells were subjected to hypoxia-reoxygenation (H/R) treatment. After treatment, cells were incubated with 200 nM MitoTracker™ Red in serum-free medium for 30 min at 37 °C in the dark. Unbound dye was removed by washing twice with pre-warmed PBS. Mitochondrial imaging was performed using a confocal laser scanning microscope (FV3000, Olympus, Japan) with excitation/emission wavelengths of 579/599 nm.

### Assessment of mitochondrial permeability transition pore (mPTP) opening

The opening of the mitochondrial permeability transition pore (mPTP) was assessed using the Mitochondrial Permeability Transition Pore Assay Kit (Beyotime, China, #C2009S) according to the manufacturer's instructions. Briefly, after the respective treatments, AML12 cells were incubated with the mPTP fluorescent probe Calcein-AM (1 μM) and CoCl_2_ (1 mM) to quench cytosolic and nuclear fluorescence. The cells were then imaged using a confocal laser scanning microscope (FV3000, Olympus, Japan).

### Measurement of mitochondrial calcium (Ca^2+^) levels

Mitochondrial calcium levels were measured using the Mitochondrial Calcium Assay Kit (Beyotime, China, #C2009S). After treatments, cells were loaded with the fluorescent mitochondrial Ca^2+^ indicator Rhod-2 AM (5 μM) at 37 °C for 30 min. Following dye loading and washing, the fluorescence intensity (Ex/Em = 552/581 nm) was measured using a microplate reader (Synergy H1, BioTek, USA). The relative mitochondrial Ca^2+^ level was expressed as the fold change relative to the control group after background subtraction.

### Analysis of mitochondrial ultrastructure by transmission electron microscopy (TEM)

For the evaluation of mitochondrial ultrastructure, AML12 cells from different treatment groups were fixed with 2.5% glutaraldehyde in 0.1 M phosphate buffer (pH 7.4) overnight at 4 °C. After washing, the cells were post-fixed with 1% osmium tetroxide, dehydrated through a graded ethanol series, and embedded in epoxy resin. Ultrathin sections (70–90 nm) were cut using an ultramicrotome (Leica, Germany), stained with uranyl acetate and lead citrate, and examined under a transmission electron microscope (HT7800, Hitachi, Japan). Mitochondrial morphology was observed and imaged at magnifications of 8000 × and 20,000 × .

### Statistical analysis

Quantitative data are presented as the mean ± standard deviation (SD) from at least three independent biological replicates. The normality of the data distribution was assessed using the Shapiro–Wilk test. For comparisons between two groups, statistical significance was determined using an unpaired two-tailed Student's *t*-test for normally distributed data or the Mann–Whitney *U* test for non-parametric data. Multiple group comparisons were analyzed by one-way analysis of variance (ANOVA) followed by Tukey's post-hoc test for parametric data or the Kruskal–Wallis test with Dunn's correction for non-parametric data. All statistical analyses were performed using GraphPad Prism software (version 9.0, GraphPad Software, San Diego, USA). *P* value < 0.05 was considered statistically significant.

## Results

### Hepatic steatosis exacerbates ischemia–reperfusion injury and promotes apoptotic activation

**Figure 1:**
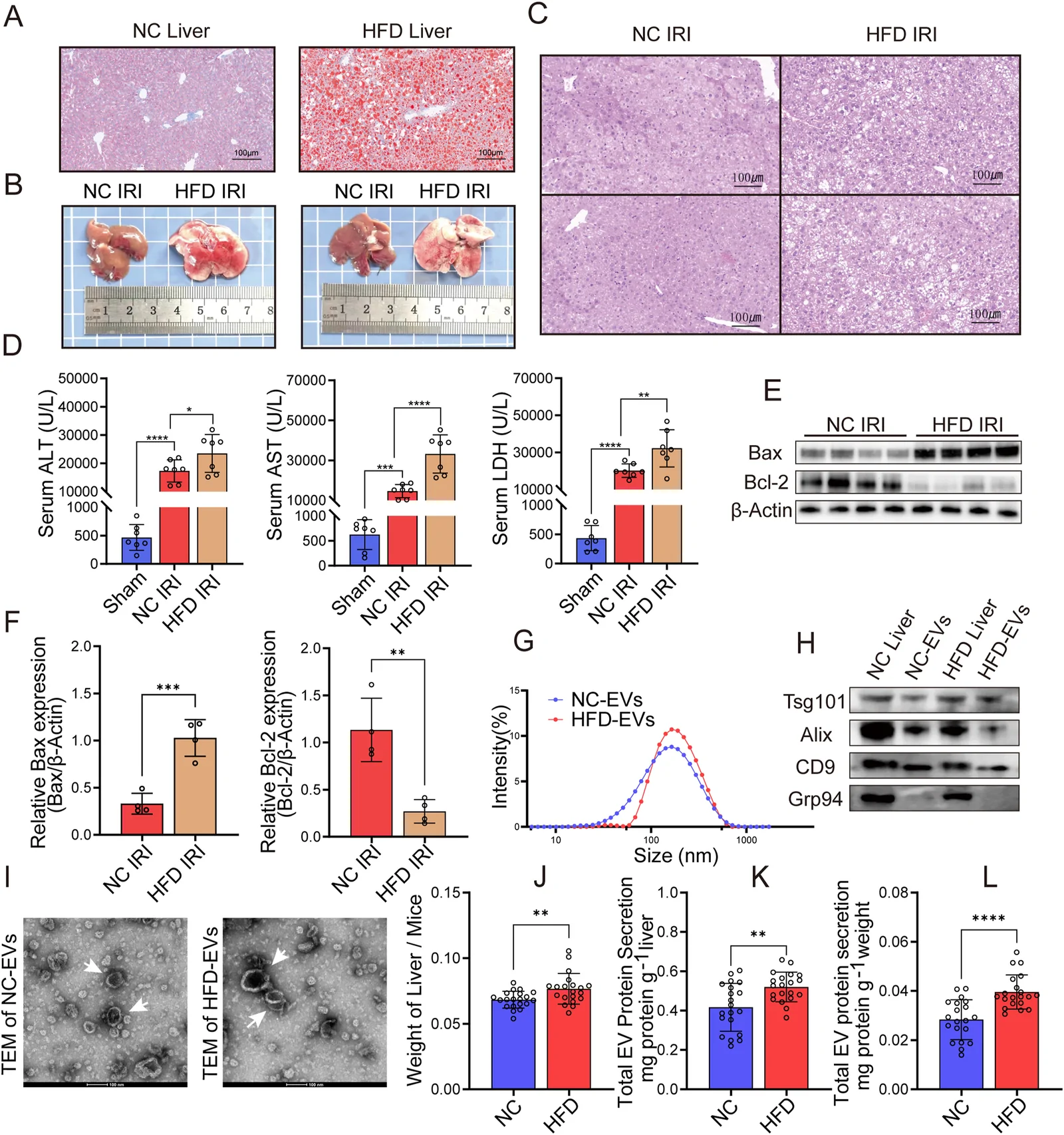
Hepatic steatosis exacerbates ischemia–reperfusion injury and enhances the production of liver-derived extracellular vesicles. Mice were fed a normal chow (NC) or high-fat diet (HFD) for 12 weeks to induce hepatic steatosis, followed by sham surgery or hepatic ischemia–reperfusion injury (HIRI). **(A)** Representative Oil Red O staining of liver sections from the NC-fed and HFD-fed mice, confirming substantial lipid accumulation in steatotic livers. **(B)** Gross morphological appearance of livers from NC IRI and HFD IRI groups, showing increased necrotic areas and congestion in steatotic livers post-IRI. **(C)** Representative hematoxylin and eosin-stained liver sections from the NC IRI and HFD IRI groups, demonstrating severe hepatocellular necrosis, inflammatory infiltration, and architectural disruption in HFD IRI livers. **(D)** Serum levels of alanine aminotransferase (ALT), aspartate aminotransferase (AST), and lactate dehydrogenase (LDH) in the indicated groups (*n* = 7). **(E, F)** Western blot analysis (E) and corresponding quantification (F) of Bcl-2 and Bax protein expression in liver tissues from the indicated groups. **(G)** Size distribution profile of extracellular vesicles (EVs) isolated from normal (NC-EVs) and steatotic (HFD-EVs) livers, as determined by dynamic light scattering (DLS). **(H)** Immunoblot analysis of canonical EV markers (CD9, Tsg101, Alix) and the negative control Grp94 in NC-EVs and HFD-EVs. **(I)** Representative transmission electron microscopy (TEM) images of NC-EVs and HFD-EVs, showing typical cup-shaped morphology. **(J)** Liver-to-body weight ratio of NC-fed and HFD-fed mice (*n* = 20). **(K, L)** Quantification of EV protein yield normalized to liver weight (K) or body weight (L) (*n* = 20). Data are presented as the mean ± SD. ∗*p* < 0.05, ∗∗*p* < 0.01, ∗∗∗*p* < 0.001, ∗∗∗∗*p* < 0.0001 (one-way ANOVA with Tukey's post-hoc test for D, F; unpaired two-tailed Student's *t*-test for J–L).

To define the impact of hepatic steatosis on susceptibility to IRI, we established a murine model of fatty liver disease via prolonged high-fat diet (HFD) feeding. Histological assessment of Oil Red O-stained liver sections confirmed massive lipid accumulation in HFD-fed mice compared with normal chow (NC)-fed controls, verifying the successful induction of steatosis (Fig. 1A).

Following standardized HIRI challenge, steatotic livers exhibited markedly aggravated injury. Gross inspection revealed more extensive necrotic areas and severe sinusoidal congestion in HFD-fed mice relative to lean controls (Fig. 1B). Consistent with this, hematoxylin and eosin-stained sections demonstrated substantially heightened hepatocellular necrosis, enhanced inflammatory infiltration, and architectural disarray in steatotic livers post-HIRI (Fig. 1C). Biochemical analysis further confirmed this exaggerated injury phenotype, as evidenced by significantly higher serum levels of ALT, AST, and LDH in HFD-fed mice than in NC-fed controls following HIRI (Fig. 1D).

Given the central role of apoptotic cell death in HIRI pathogenesis, we investigated whether steatosis exacerbated injury through this pathway. Immunoblot analysis of liver lysates showed a significant reduction in the anti-apoptotic protein Bcl-2 and a concurrent increase in the pro-apoptotic protein Bax in steatotic livers after HIRI (Fig. 1E and F). This pronounced shift in the Bcl-2/Bax balance toward apoptosis promotion provides a molecular correlate for the observed sensitivity of steatotic livers to ischemic insult.

Together, these data demonstrate that hepatic steatosis significantly worsens HIRI outcomes and is associated with enhanced activation of apoptotic signaling.

### Characterization of liver-derived extracellular vesicles reveals enhanced production in steatosis

To investigate the potential contribution of EVs to the heightened vulnerability of steatotic livers to HIRI, we isolated EVs from the liver tissues of mice fed either a normal chow (NC-EVs) or a high-fat diet (HFD-EVs) using differential ultracentrifugation.

The isolated vesicles underwent rigorous characterization to confirm their identity and purity. Dynamic light scattering (DLS) analysis indicated that both NC-EVs and HFD-EVs exhibited a size distribution profile characteristic of small EVs, with no significant difference in mean particle diameter between the groups (Fig. 1G). Morphological assessment by TEM confirmed the presence of intact, cup-shaped vesicles bounded by a lipid bilayer, consistent with typical EV ultrastructure (Fig. 1I). Furthermore, immunoblot analysis validated the enrichment of established EV markers (CD9, Tsg101, Alix) and the absence of the endoplasmic reticulum resident protein Grp94, confirming minimal contamination by cellular organelles (Fig. 1H).

Notably, quantification of EV protein content using the BCA assay revealed a significantly higher protein concentration in HFD-EVs compared to NC-EVs, whether normalized to liver weight or total tissue protein (Fig. 1J–L). This finding suggests that the state of hepatic steatosis is associated with an increased release of EVs and/or an alteration in their protein cargo loading capacity.

In summary, we successfully isolated and characterized liver-derived EVs, demonstrating that steatotic livers exhibit a marked increase in EV production. This quantitative shift in EV release provides a potential mechanistic link through which fatty livers may actively modulate the hepatic microenvironment, potentially contributing to the exacerbation of HIRI.

### Proteomic profiling identifies a pathogenic cargo in HFD-EVs, enriched in ferroptotic mediators and deficient in mitochondrial metabolic components

**Figure 2:**
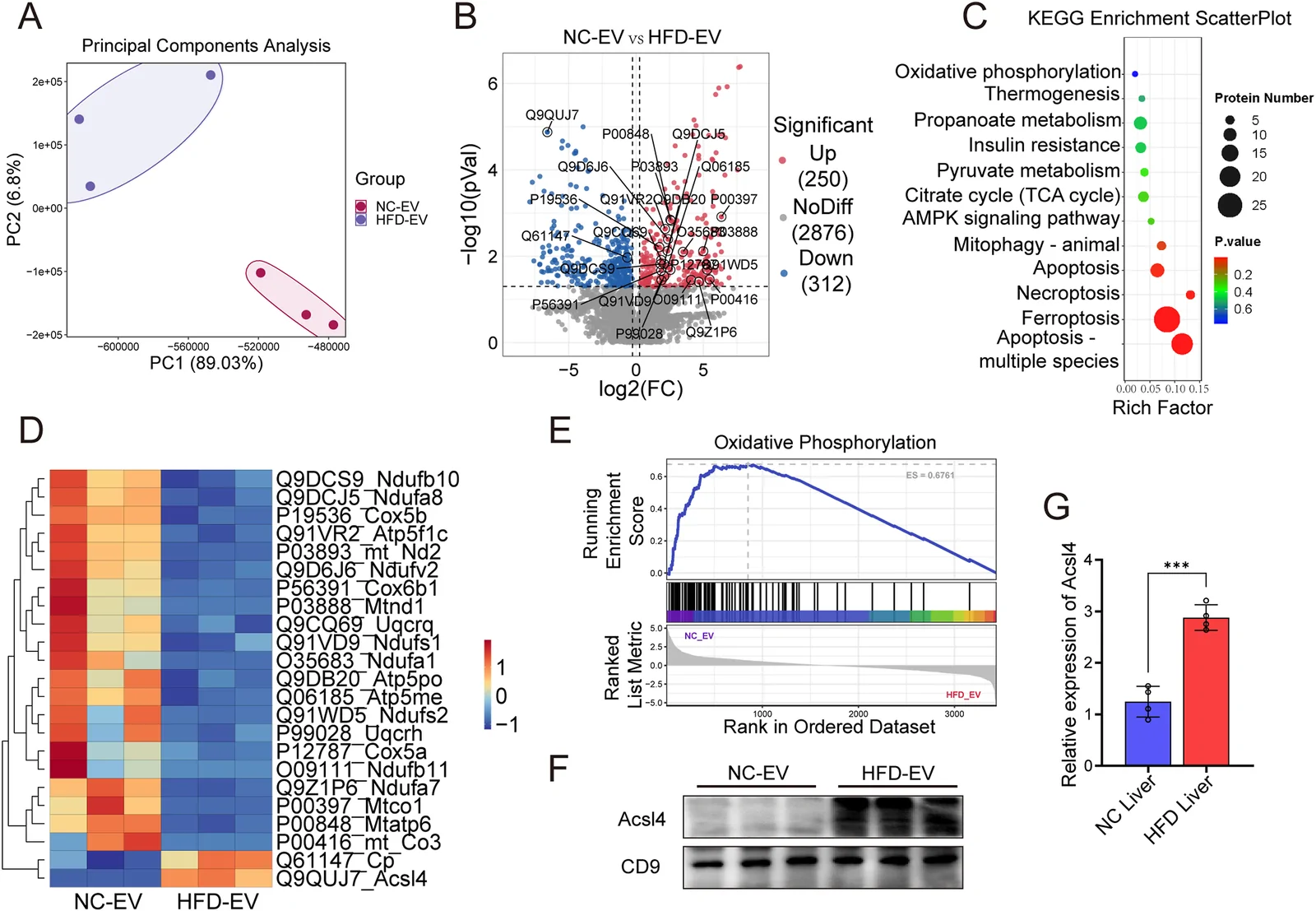
Proteomic profiling of HFD-EVs reveals a pathogenic cargo enriched in ferroptotic mediators and deficient in mitochondrial metabolic components. **(A)** Principal component analysis (PCA) plot showing distinct proteomic profiles of NC-EVs and HFD-EVs. **(B)** Volcano plot displaying differentially expressed proteins between NC-EVs and HFD-EVs. **(C)** KEGG pathway enrichment analysis of proteins in HFD-EVs compared to NC-EVs (pathways were selected from the complete KEGG enrichment analysis based on statistical significance and relevance to mitochondrial function and cell death mechanisms). **(D)** Heatmap of selected differentially expressed proteins related to ferroptosis (*e.g.*, ACSL4) and mitochondrial oxidative phosphorylation (OXPHOS) in NC-EVs and HFD-EVs. **(E)** Gene Set Enrichment Analysis (GSEA) plot demonstrating significant negative enrichment of the OXPHOS pathway in HFD-EVs compared to NC-EVs. **(F)** Western blot analysis validating the increased protein level of ACSL4 in HFD-EVs relative to NC-EVs. **(G)** qPCR analysis of Acsl4 mRNA expression in liver tissues from NC-fed and HFD-fed mice (*n* = 4). Data are presented as the mean ± SD. ∗*p* < 0.05, ∗∗*p* < 0.01 (unpaired two-tailed Student's *t*-test).

To uncover the molecular mechanisms by which HFD-EVs exacerbate HIRI, we conducted a comparative proteomic analysis of EVs isolated from steatotic (HFD-EVs) and normal (NC-EVs) livers. Unsupervised principal component analysis (PCA) revealed a clear segregation between the proteomic profiles of HFD-EVs and NC-EVs, indicating a fundamental remodeling of the EV proteome in steatosis (Fig. 2A). Quantitative profiling identified 562 differentially expressed proteins, with 312 up-regulated and 250 down-regulated in HFD-EVs (Fig. 2B).

Strikingly, focused analysis of the altered proteins revealed a coherent pathogenic signature. Heatmap visualization highlighted a significant up-regulation of pro-ferroptotic proteins, most notably ACSL4, alongside a marked down-regulation of multiple core subunits of the mitochondrial oxidative phosphorylation (OXPHOS) system (Fig. 2D). This inverse pattern suggested that HFD-EVs are packaged with cargo poised to simultaneously induce lipid peroxidation stress and compromise cellular energy metabolism.

We next sought to determine the broader pathway-level implications of these changes. KEGG enrichment analysis of all differentially expressed proteins confirmed that up-regulated proteins in HFD-EVs were significantly associated with ferroptosis and apoptosis, while down-regulated proteins were prominently enriched in metabolic pathways, particularly oxidative phosphorylation (Fig. 2C). To specifically and rigorously assess the suppression of the mitochondrial energy pathway, we performed Gene Set Enrichment Analysis (GSEA). This analysis confirmed a significant negative enrichment score for the OXPHOS pathway in HFD-EVs, validating a coordinated loss of mitochondrial metabolic components (Fig. 2E).

Guided by the proteomic findings, we validated the expression of pivotal ferroptosis regulators. Immunoblot analysis confirmed a robust increase in ACSL4 in HFD-EVs compared to NC-EVs (Fig. 2F). Consistent with this, qPCR analysis of liver tissues showed significant up-regulation of Acsl4 mRNA in HFD-fed mice (Fig. 2G), indicating that the altered packaging of these molecules into EVs reflects transcriptional changes occurring in the steatotic liver.

In summary, our proteomic and subsequent validation studies demonstrate that HFD-EVs carry a distinct and pathogenic cargo repertoire. This repertoire is characterized by an acquired pro-ferroptotic potential, driven by ACSL4 enrichment, and a loss of proteins essential for mitochondrial respiration. This dual insult likely underlies the enhanced potency of HFD-EVs in disrupting mitochondrial integrity and triggering cell death during HIRI.

### HFD-EVs potentiate HIRI by amplifying apoptosis and inflammation

**Figure 3:**
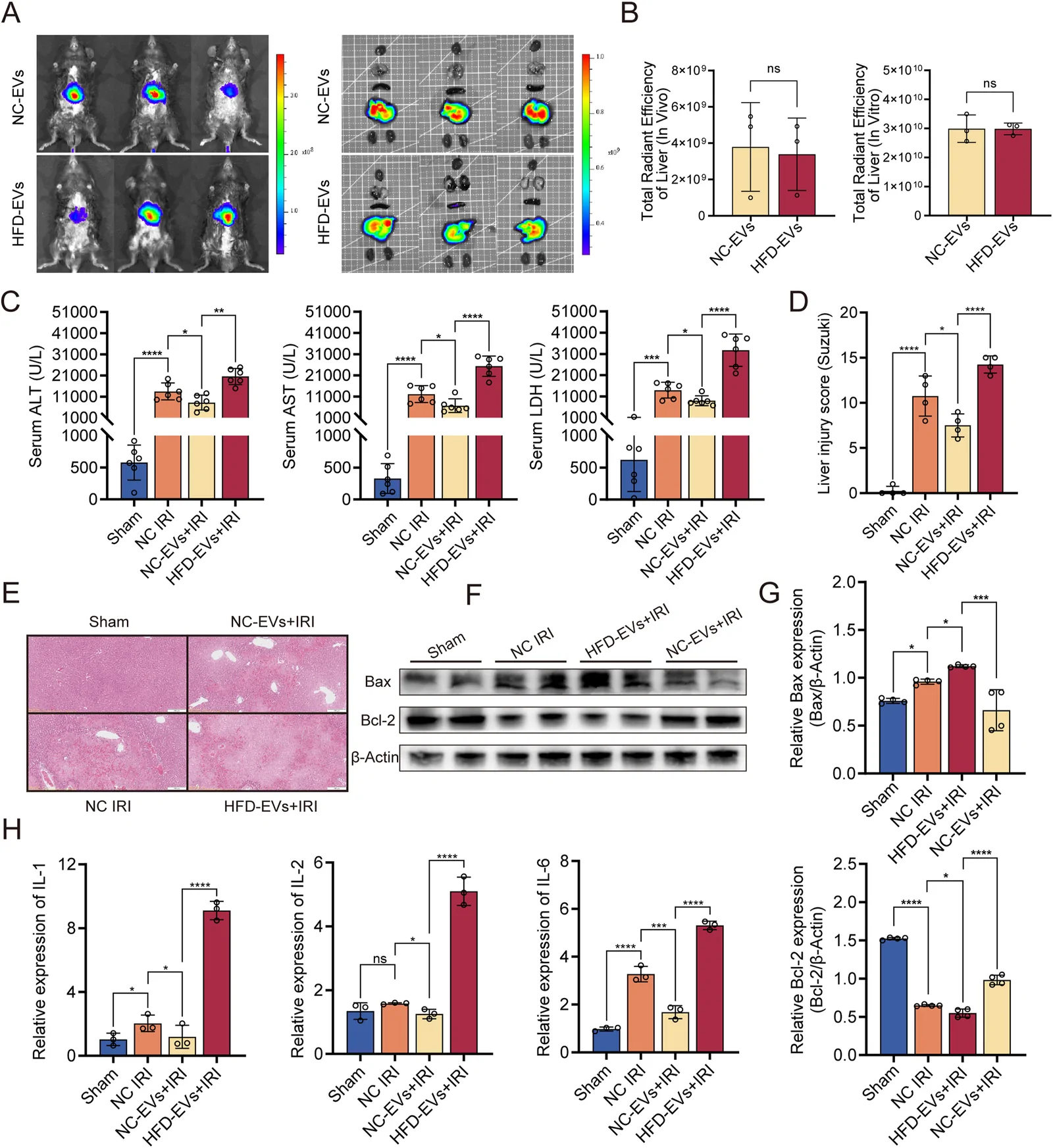
Administration of HFD-EVs exacerbates hepatic ischemia–reperfusion injury by amplifying apoptosis and inflammation in lean mice. Lean mice were intravenously injected with PBS, extracellular vesicles from normal chow-fed mice (NC-EVs, 20 mg/kg), or extracellular vesicles from high-fat diet-fed mice (HFD-EVs, 20 mg/kg) 24 h prior to sham surgery or hepatic ischemia–reperfusion injury (IRI). **(A)** Representative *in vivo* and *ex vivo* fluorescence images showing the biodistribution of DIR-labeled NC-EVs and HFD-EVs at 24 h post-injection. **(B)** Quantitative analysis of DIR signal intensity in the liver, confirming comparable hepatic accumulation of NC-EVs and HFD-EVs (*n* = 3). **(C)** Serum levels of ALT, AST, and LDH in the indicated groups at 6 h post-reperfusion (*n* = 6). **(D)** Suzuki scores quantifying the severity of liver injury based on histological analysis (*n* = 4). **(E)** Representative hematoxylin and eosin-stained liver sections from the indicated groups. **(F)** Western blot analysis of Bax and Bcl-2 protein expression in liver tissues from the indicated groups. **(G)** Quantitative analysis of the Bax/Bcl-2 ratio derived from (F) (*n* = 4). **(H)** qPCR analysis of pro-inflammatory cytokine (IL-1, IL-2, IL-6) mRNA expression in liver tissues (*n* = 3). Data are presented as the mean ± SD. ∗*p* < 0.05, ∗∗*p* < 0.01, ∗∗∗*p* < 0.001, ∗∗∗∗*p* < 0.0001 (one-way ANOVA with Tukey's post-hoc test).

To functionally validate the pathogenic role of steatotic liver-derived EVs, we intravenously administered HFD-EVs or NC-EVs (20 mg/kg) to lean mice 24 h prior to inducing HIRI. *In vivo* tracking confirmed comparable hepatic accumulation of both EV types (Fig. 3A and B), indicating that the functional divergence was not attributable to differences in biodistribution.

Strikingly, pretreatment with EVs from different sources exerted opposing effects on HIRI severity. Mice receiving HFD-EVs sustained the most severe liver damage, as evidenced by significantly elevated serum levels of ALT, AST, and LDH (Fig. 3C), extensive panlobular necrosis upon hematoxylin and eosin staining (Fig. 3E), and the highest Suzuki injury scores (Fig. 3D). In contrast, administration of NC-EVs significantly attenuated IRI-induced liver injury compared to the NC IRI group, suggesting a protective role of EVs derived from healthy livers.

At the molecular level, Western blot analysis revealed that this contrasting phenotype was associated with differential regulation of the apoptosis pathway. The HFD-EVs + IRI group exhibited a significant increase in the pro-apoptotic protein Bax and a decrease in the anti-apoptotic protein Bcl-2, resulting in a markedly elevated Bax/Bcl-2 ratio compared to all other groups (Fig. 3F and G). Notably, NC-EV pretreatment showed a tendency to reverse the IRI-induced Bax/Bcl-2 imbalance, aligning with its protective role at the histological level.

Furthermore, the inflammatory response was significantly modulated. qPCR analysis demonstrated that HFD-EV pretreatment led to a substantial upregulation of the mRNA expression of key pro-inflammatory cytokines (IL-1, IL-2, IL-6) in IRI-injured livers. This pro-inflammatory effect was not observed in the NC-EVs + IRI group, which exhibited cytokine levels comparable to or lower than those in the NC IRI group (Fig. 3H).

In summary, these *in vivo* data demonstrate a critical functional dichotomy: while NC-EVs from healthy livers appear to confer a protective effect against HIRI, HFD-EVs act as a potent pathological vector that exacerbates injury by amplifying apoptotic signaling and potentiating a robust pro-inflammatory response.

### HFD-EVs trigger mitochondrial permeability transition and ferroptotic signaling to amplify hepatocyte death

**Figure 4:**
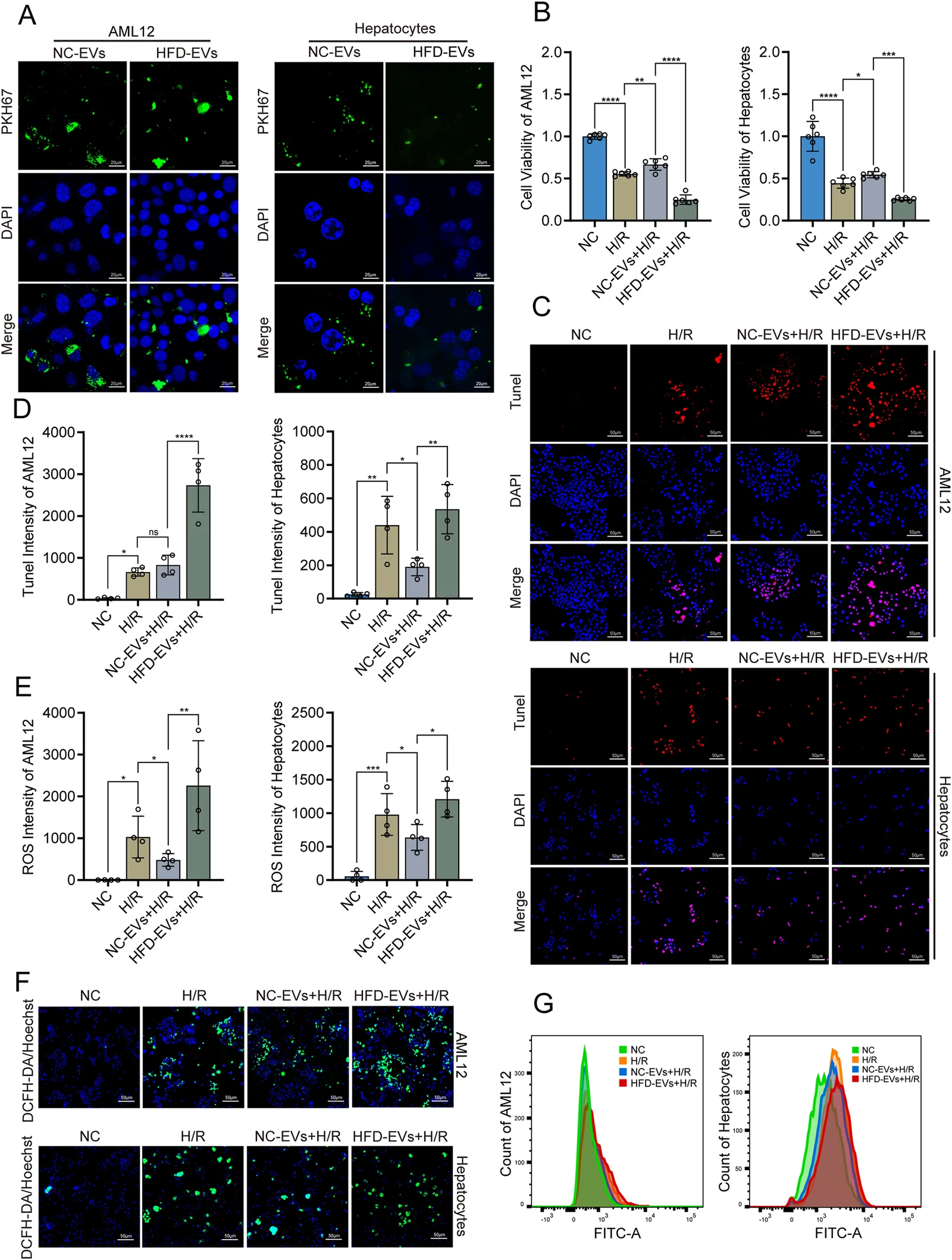
HFD-EVs sensitize hepatocytes to hypoxia-reoxygenation injury by promoting oxidative stress and apoptosis *in vitro*. AML12 cells and primary mouse hepatocytes were pretreated with PBS, NC-EVs (100 μg/mL), or HFD-EVs (100 μg/mL) for 24 h, followed by hypoxia-reoxygenation (H/R) insult. **(A)** Confocal microscopy images showing efficient cellular uptake of PKH67-labeled (green) NC-EVs and HFD-EVs by AML12 cells and primary hepatocytes. Nuclei are stained with DAPI (blue). **(B)** Cell viability assessed by CCK-8 assay in AML12 cells and primary hepatocytes after the indicated treatments (*n* = 6). **(C, D)** Representative TUNEL staining (red) images (C) and corresponding quantification of fluorescence intensity (D) demonstrating apoptosis in AML12 cells and primary hepatocytes. Nuclei are stained with DAPI (blue) (*n* = 4). **(E)** Representative flow cytometry histograms of DCFH-DA fluorescence intensity in AML12 cells and primary hepatocytes, reflecting intracellular ROS levels. **(F)** Representative fluorescence microscopy images of intracellular ROS levels detected by DCFH-DA staining (green) in AML12 cells and primary hepatocytes. **(G)** Representative flow cytometry histograms of DCFH-DA fluorescence intensity in AML12 cells and primary hepatocytes, reflecting the distribution of intracellular ROS levels across different treatment groups. Data are presented as the mean ± SD. ∗*p* < 0.05, ∗∗*p* < 0.01, ∗∗∗*p* < 0.001, ∗∗∗∗*p* < 0.0001 (one-way ANOVA with Tukey's post-hoc test).

To dissect the mechanistic cascade underlying the opposing effects of EVs observed *in vivo*, we employed *in vitro* hypoxia-reoxygenation (H/R) models using AML12 cells and primary hepatocytes. Cellular uptake assays confirmed the efficient and comparable internalization of both NC-EVs and HFD-EVs (Fig. 4A). Mirroring our *in vivo* findings, pretreatment with EVs dictated cell fate upon H/R insult. Notably, NC-EVs significantly improved cell viability after H/R compared to the H/R alone group, underscoring their intrinsic protective property. In stark contrast, HFD-EV pretreatment potently sensitized hepatocytes to H/R-induced death, resulting in the most substantial reduction in viability (Fig. 4B). This dichotomous effect on cytotoxicity was further corroborated by TUNEL staining, which showed a markedly higher rate of apoptosis in the HFD-EVs + H/R group, while the NC-EVs + H/R group exhibited significantly less apoptosis than the H/R control (Fig. 4C and D).

We next probed the signaling events leading to this differential survival. Quantitative analysis by flow cytometry and qualitative assessment by fluorescence microscopy consistently revealed that HFD-EV co-incubation significantly intensified H/R-induced oxidative stress, as evidenced by elevated intracellular ROS levels. This pro-oxidant effect was substantially attenuated by NC-EV pretreatment (Fig. 4E–G).

**Figure 5:**
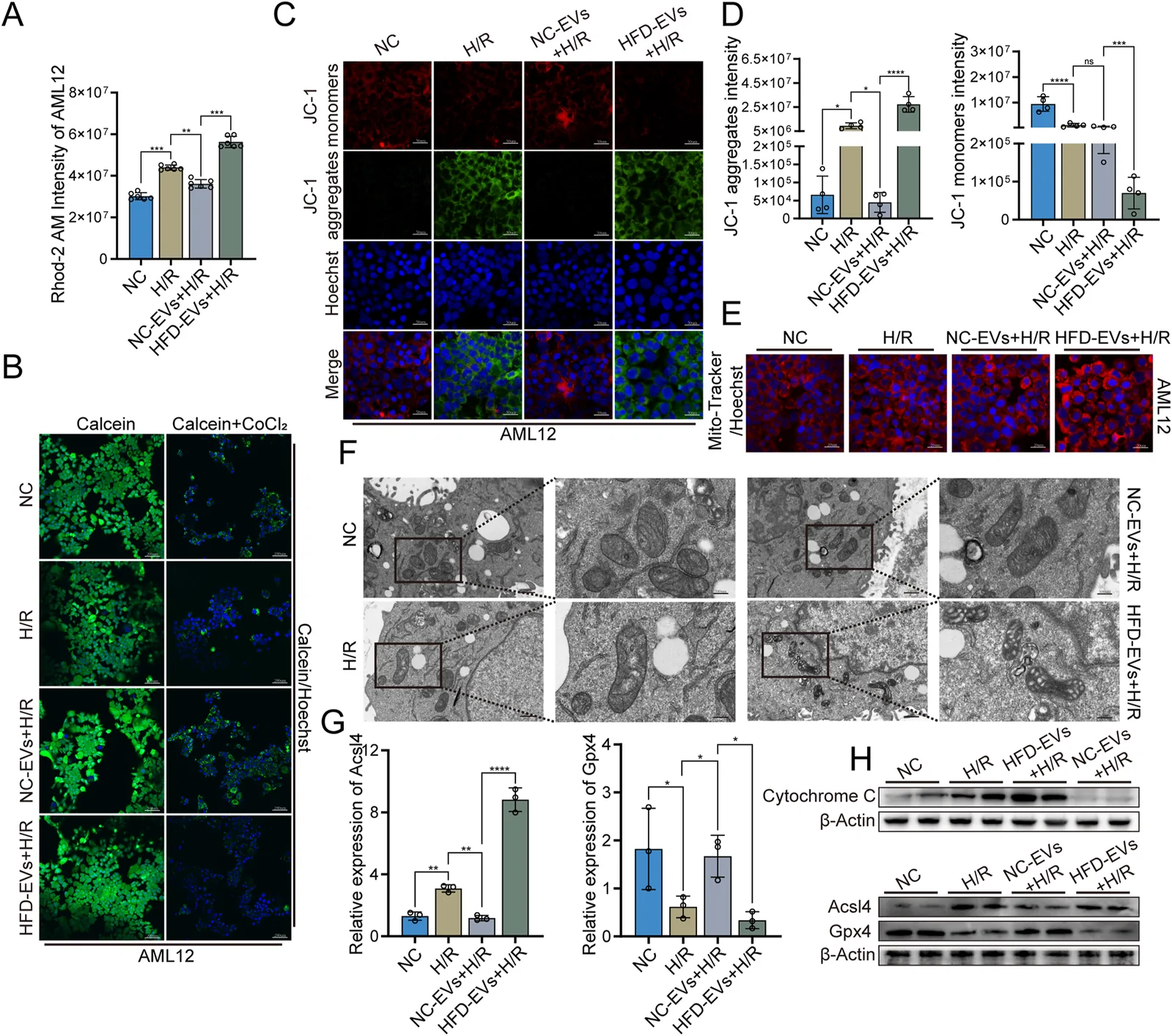
HFD-EVs trigger mitochondrial permeability transition and ferroptotic signaling to amplify hepatocyte death. AML12 cells were pretreated with NC-EVs or HFD-EVs (100 μg/mL) for 24 h, followed by hypoxia-reoxygenation (H/R) insult. **(A)** Quantitative analysis of mitochondrial Ca^2+^ levels (*n* = 6). **(B)** Representative fluorescence images assessing mitochondrial permeability transition pore (mPTP) opening. **(C)** Representative confocal microscopy images of mitochondrial membrane potential (ΔΨm) assessed by JC-1 staining. Red fluorescence (J-aggregates) indicates polarized/healthy mitochondria; green fluorescence (J-monomers) indicates depolarized/dysfunctional mitochondria. **(D)** Quantitative analysis of JC-1 red fluorescence intensity (right panel, indicating healthy mitochondria) and green fluorescence intensity (left panel, indicating depolarized mitochondria) from (C) (*n* = 4). **(E)** Representative confocal microscopy images of mitochondrial morphology visualized by MitoTracker™ Red CMXRos staining. **(F)** Representative transmission electron microscopy (TEM) images showing mitochondrial ultrastructure in hepatocytes under the indicated conditions. **(G)** Quantitative analysis of ACSL4, GPX4, and cytosolic cytochrome c protein expression from Western blot assays (*n* = 3). **(H)** Original Western blot images showing the protein levels of ACSL4, GPX4, and cytochrome c in the cytosolic fraction. Data are presented as the mean ± SD. ∗*p* < 0.05, ∗∗*p* < 0.01, ∗∗∗*p* < 0.001, ∗∗∗∗*p* < 0.0001 (one-way ANOVA with Tukey's post-hoc test).

The culmination of this oxidative burden was severe mitochondrial dysfunction. Assessment of mitochondrial Ca^2+^ flux revealed a substantial overload specifically in the HFD-EVs + H/R group (Fig. 5A). This was directly linked to the opening of the mitochondrial permeability transition pore (mPTP), as quantified by a significant increase in fluorescence intensity (Fig. 5B). Consistent with mPTP opening, JC-1 staining demonstrated a severe loss of mitochondrial membrane potential (ΔΨm) in HFD-EVs-treated cells, which was markedly preserved by NC-EV pretreatment (Fig. 5C and D). We next assessed the impact on mitochondrial network integrity. MitoTracker™ staining revealed that H/R stress alone induced mild mitochondrial fragmentation in hepatocytes. However, pretreatment with HFD-EVs dramatically exacerbated this phenotype, resulting in severe fragmentation and punctate morphology of the mitochondrial network. In clear contrast, NC-EV pretreatment effectively maintained a more tubular and interconnected mitochondrial morphology, similar to normoxic controls (Fig. 5E). Most compellingly, visualization of mitochondrial ultrastructure by TEM provided direct morphological evidence of these functional changes. Hepatocytes subjected to H/R alone showed moderate swelling and cristae disorganization. Strikingly, HFD-EVs + H/R treatment induced the most severe pathology, including marked swelling, cristae fragmentation, and outer membrane rupture. Conversely, NC-EV pretreatment effectively mitigated these ultrastructural aberrations (Fig. 5F).

This catastrophic mitochondrial failure was the pivotal event triggering apoptotic execution, as evidenced by the robust release of cytochrome c into the cytosol in the HFD-EVs + H/R group (Fig. 5H). Crucially, we investigated whether this process was driven by the pro-ferroptotic signature identified in the HFD-EV proteome. Indeed, HFD-EV treatment led to a significant up-regulation of ACSL4 and down-regulation of GPX4 at both protein and mRNA levels in recipient hepatocytes (Fig. 5G and H), thereby instigating a ferroptotic signaling cascade that precipitated the observed mitochondrial permeability transition and apoptosis.

In summary, these *in vitro* data delineate a coherent pathogenic pathway: HFD-EVs instigate oxidative stress, which converges with ACSL4/GPX4-mediated ferroptotic signaling to drive mitochondrial permeability transition, ultrastructural collapse, and culminating in cytochrome c release and amplified cell death. Conversely, NC-EVs confer protection by stabilizing mitochondrial integrity against H/R insult.

### Ferroptosis inhibition abrogates HFD-EVs-induced mitochondrial damage and cytotoxicity

To definitively establish that the transfer of functional ACSL4 by HFD-EVs activates a ferroptosis-driven pathway leading to mitochondrial dysfunction and cell death, we conducted a series of rescue experiments using the specific ferroptosis inhibitor Ferrostatin-1 (Fer-1) in AML12 cells subjected to hypoxia-reoxygenation (H/R).

**Figure 6:**
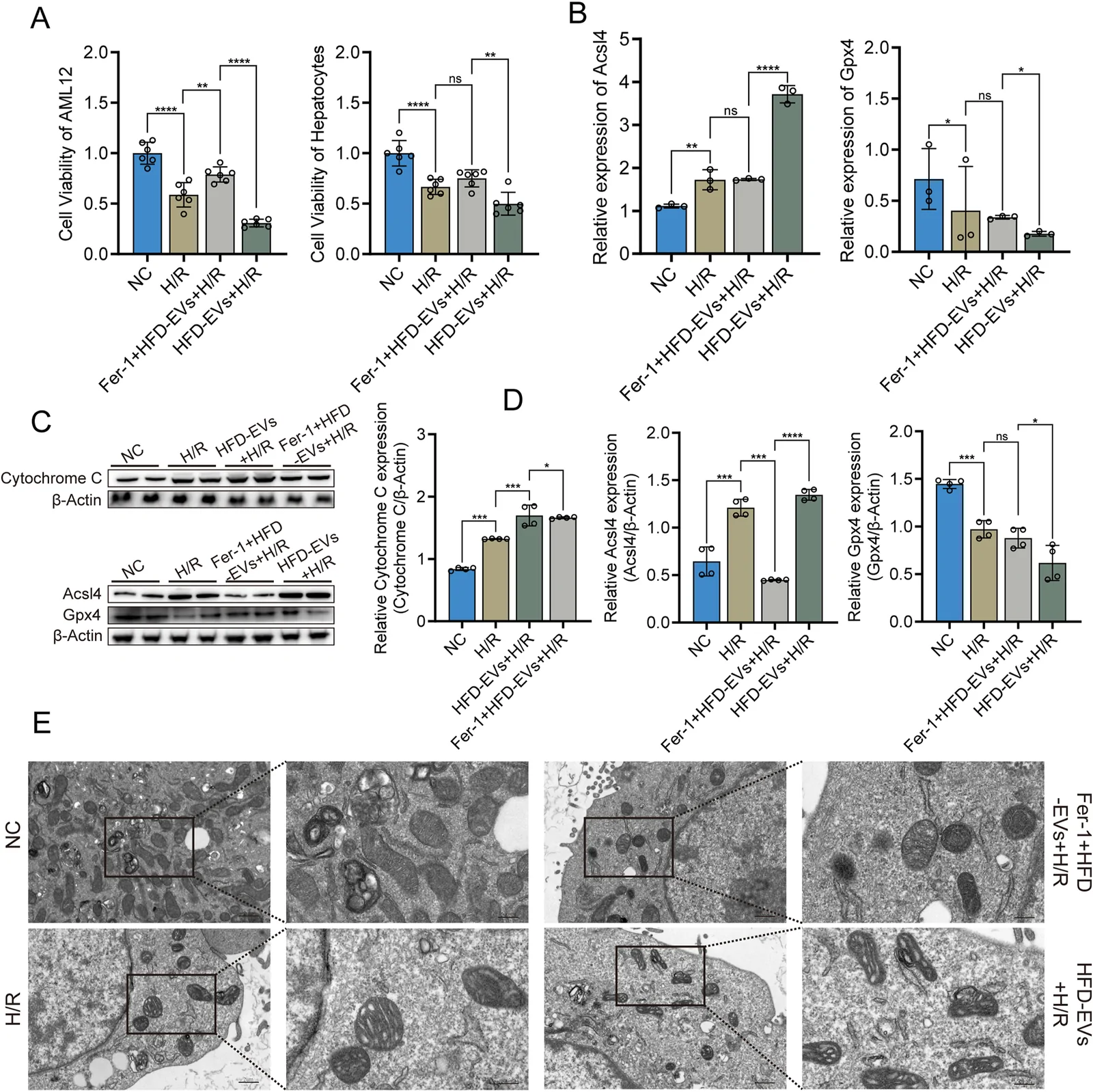
Inhibition of ferroptosis abrogates HFD-EVs-induced mitochondrial damage and cytotoxicity. AML12 cells were pretreated with HFD-EVs (100 μg/mL) in the presence or absence of the ferroptosis inhibitor Ferrostatin-1 (Fer-1, 2 μM) for 24 h, followed by hypoxia-reoxygenation (H/R) insult. **(A)** Cell viability assessed by CCK-8 assay after the indicated treatments (*n* = 6). **(B)** qPCR analysis of Acsl4 and Gpx4 mRNA expression in AML12 cells (*n* = 3). **(C)** Western blot images showing the protein levels of ACSL4, GPX4, and cytochrome c in the cytosolic fraction. **(D)** Quantitative analysis of ACSL4, GPX4, and cytosolic cytochrome c protein expression from (C) (*n* = 4). **(E)** Representative transmission electron microscopy (TEM) images of mitochondrial ultrastructure in AML12 cells under the indicated conditions. Data are presented as the mean ± SD. ∗*p* < 0.05, ∗∗*p* < 0.01, ∗∗∗*p* < 0.001, ∗∗∗∗*p* < 0.0001 (one-way ANOVA with Tukey's post-hoc test).

Strikingly, co-incubation with Fer-1 significantly restored cell viability in the HFD-EVs + H/R group, as quantified by CCK-8 assay (Fig. 6A). This functional rescue provided direct evidence that ferroptosis constitutes a major executable mechanism underlying HFD-EVs-induced cytotoxicity.

This functional preservation was underpinned by a complete normalization of the ferroptotic signaling cascade at the molecular level. Immunoblot and qPCR analyses demonstrated that Fer-1 treatment effectively reversed the HFD-EVs-mediated dysregulation of key ferroptotic mediators, abrogating the up-regulation of ACSL4 and restoring the expression of GPX4 to baseline levels at both the protein and transcript levels (Fig. 6B–D). These findings conclusively show that the detrimental effects of HFD-EVs are mechanistically dependent on the activation of the ACSL4/GPX4 axis within the ferroptosis pathway.

We next investigated whether halting ferroptosis could prevent the ensuing mitochondrial damage and apoptotic execution. TEM examination of mitochondrial ultrastructure provided compelling structural evidence. While HFD-EVs + H/R treatment induced severe mitochondrial pathology—including marked swelling, cristae fragmentation, and outer membrane rupture—co-treatment with Fer-1 conferred remarkable protection, preserving mitochondrial architecture with clearly defined cristae and intact membranes (Fig. 6E). Consistent with this structural preservation, the robust release of cytochrome c into the cytosol observed in the HFD-EVs + H/R group was effectively suppressed by Fer-1 co-treatment (Fig. 6C and D).

In summary, these genetic and pharmacological rescue experiments delineate a coherent pathogenic sequence: HFD-EVs deliver ACSL4 to recipient hepatocytes, instigating a ferroptotic cascade that acts as an upstream trigger for catastrophic mitochondrial permeability transition and structural collapse. The fact that pharmacological inhibition of ferroptosis alone is sufficient to uncouple HFD-EV exposure from both mitochondrial failure and cell death provides compelling causal evidence for this mechanism.

## Discussion

HIRI remains a major obstacle in liver transplantation, with the escalating use of steatotic donor livers significantly compounding this challenge due to their heightened vulnerability.^14^^,^^15^ While the detrimental role of steatosis is clinically recognized, the precise intercellular mechanisms orchestrating this susceptibility are not fully elucidated. Our study unveils a novel paradigm by identifying HFD-EVs as critical mediators that transmit a pro-ferroptotic signal to recipient hepatocytes, thereby initiating a mitochondrial apoptosis cascade and amplifying HIRI.

The most salient finding of our work is the proteomic reprogramming of HFD-EVs towards a ferroptosis-promoting phenotype. We identified ACSL4, a key enzyme that esterifies polyunsaturated fatty acids into membranes and thereby primes them for peroxidation,^16^ as being markedly enriched in HFD-EVs. Concurrently, HFD-EVs were deficient in core components of the mitochondrial oxidative phosphorylation system. This dual cargo—equipping recipient cells with the “fuel” for lipid peroxidation while depriving them of efficient energy production—creates a perfect storm, predisposing hepatocytes to ferroptotic death upon the additional oxidative burst of H/R. This finding provides a mechanistic explanation for the known sensitivity of steatotic livers to ferroptosis and positions EVs as the delivery vehicles of this pathogenic signal.^17^

Functionally, we demonstrated that HFD-EVs, but not NC-EVs, act as a potent pathological vector that exacerbates HIRI in lean recipients. This effect was multimodal: HFD-EVs coordinatedly amplified mitochondrial apoptosis and pro-inflammatory responses *in vivo*. The *in vitro* data further dissected the precise sequence of events: upon internalization, HFD-EVs instigate oxidative stress, which converges with the delivered ACSL4 to drive an iron-dependent, GPX4-suppressed ferroptotic process. Crucially, we established that this ferroptotic stress is not an endpoint but a critical upstream trigger. It directly precipitates mitochondrial permeability transition (mPTP opening), as evidenced by calcium overload, loss of membrane potential, and the profound ultrastructural damage captured by TEM. This mitochondrial catastrophe culminates in the release of cytochrome c, executing the final apoptotic program. The functional dichotomy between HFD-EVs and NC-EVs underscores that the EV cargo, rather than the vesicle itself, dictates the pathological outcome, highlighting the specific role of the steatotic microenvironment in reprogramming EV function.

The causal role of ferroptosis in this cascade was definitively established by our rescue experiments. The fact that pharmacological inhibition of ferroptosis with Ferrostatin-1 alone was sufficient to abrogate HFD-EVs-induced mitochondrial damage, cytochrome c release, and cell death provides compelling evidence that ferroptosis is the linchpin mechanism. This not only strengthens our proposed pathway but also pinpoints the ACSL4-ferroptosis axis as a promising therapeutic target. Strategies aimed at inhibiting EV biogenesis in steatotic donors (*e.g.*, using GW4869^18^, ^19^, ^20^) or directly neutralizing the pro-ferroptotic cargo of HFD-EVs could potentially “de-risk” marginal grafts, expanding the pool of useable livers.

Our study has several limitations. Firstly, the reliance on a murine model necessitates future validation in human steatotic liver samples or *ex vivo* perfusion models to confirm clinical translatability. Secondly, while we identified ACSL4 as a key component, the cargo of HFD-EVs is complex, and the potential contributions of other biomolecules (*e.g.*, lipids, miRNAs) to the observed phenotype warrant further investigation. Finally, the interaction of HFD-EVs with non-parenchymal cells (*e.g.*, Kupffer cells), which could indirectly exacerbate hepatocyte injury, remains an important area for future research.

In conclusion, we have delineated a novel mechanism by which steatotic livers exacerbate HIRI through the secretion of ACSL4-enriched EVs that trigger a ferroptosis-driven mitochondrial apoptosis cascade in hepatocytes. This work shifts the perspective from the static condition of steatosis to the dynamic, active role of EV-mediated intercellular communication in propagating injury. It provides a robust mechanistic basis for the vulnerability of fatty liver grafts and opens new avenues for therapeutic intervention by targeting the HFD-EVs/ACSL4/ferroptosis pathway to improve outcomes in liver transplantation.

## CRediT authorship contribution statement

**Hangcheng Zhao:** Writing – original draft, Investigation, Formal analysis, Data curation. **Jialing Zhao:** Writing – original draft, Visualization, Methodology, Data curation. **Danfeng Zhou:** Writing – original draft, Visualization, Investigation. **Yunguo Lei:** Software, Investigation. **Zhikun Liu:** Visualization, Validation, Resources. **Jun Chen:** Software, Investigation. **Peiyang Hu:** Project administration, Methodology. **Xiao Xu:** Writing – review & editing, Supervision, Project administration, Funding acquisition. **Haiyang Xie:** Writing – review & editing, Resources, Funding acquisition, Formal analysis. **Qiang Wei:** Supervision, Project administration, Funding acquisition.

## Ethics declaration

All experimental protocols were conducted in accordance with the institutional ethical guidelines and approved by Institutional Animal Care and Use Committee of Zhejiang Center of Laboratory Animals (ZJCLA-IACUC-200011061).

## Data availability

All relevant data supporting the findings of this study are included within the article. Additional data are available from the corresponding author upon reasonable request.

## Funding

This work was supported by grants from the 10.13039/501100012166National Key Research and Development Program of China (No. 2021YFA1100500), the Innovation Team of Hangzhou Medical College (China) (No. CXLJ202401) and the Projects of Medical and Health Technology Program in Zhejiang Province, China (No. 2026775598)

## Conflict of interests

The authors declare no competing financial or non-financial interests related to this work.
