# Supplementary material for: Steatotic liver-derived extracellular vesicles deliver ACSL4 to potentiate hepatic ischemia–reperfusion injury by orchestrating a ferroptosis-mitochondrial apoptosis cascade
Source: Genes Dis. 2025 Dec 24;14(1):102005. doi: 10.1016/j.gendis.2025.102005 (PMC13597912; doi:10.1016/j.gendis.2025.102005)
Supplement: Supplementary file 1 [file mmc1.docx]

**Table S1 List of Primers Used in the Study**

| **Primer Name** | **Forward Primer Sequence** | **Reverse Primer Sequence** |
| --- | --- | --- |
| Acsl4 | CCTTTGGCTCATGTGCTGGAAC | GCCATAAGTGTGGGTTTCAGTAC |
| Gpx4 | CCTCTGCTGCAAGAGCCTCCC | CTTATCCAGGCAGACCATGTGC |
| IL-1 | TGGACCTTCCAGGATGAGGACA | GTTCATCTCGGAGCCTGTAGTG |
| IL-6 | TACCACTTCACAAGTCGGAGGC | CTGCAAGTGCATCATCGTTGTTC |
| IL-10 | CGGGAAGACAATAACTGCACCC | CGGTTAGCAGTATGTTGTCCAGC |

**Table S2 List of Primary Antibodies Used in the Study**

| **Target Protein** | **Company** | **Host (Rabbit/Mouse)** | **Dilution Used** | **Catalog Number** |
| --- | --- | --- | --- | --- |
| Bcl-2 | Cell Signaling Technology, USA | Rabbit | 1:2000 | #3498 |
| Bax | Cell Signaling Technology, USA | Rabbit | 1:2000 | #5023 |
| CD9 | Cell Signaling Technology, USA | Rabbit | 1:2000 | #13174 |
| Alix | Cell Signaling Technology, USA | Rabbit | 1:2000 | #92880 |
| Tsg101 | Cell Signaling Technology, USA | Rabbit | 1:2000 | #72312 |
| Grp94 | ABclonal, China | Rabbit | 1:2000 | A0989 |
| Β-Actin | Proteintech, China | Mouse | 1:3000 | 66009-1-Ig |
| Acsl4 | ABclonal, China | Rabbit | 1:2000 | A20414 |
| Gpx4 | ABclonal, China | Rabbit | 1:2000 | A11243 |
